# Supplementary material for: Global floating algae blooms are expanding
Source: Nat Commun. 2025 Dec 7;17:612. doi: 10.1038/s41467-025-66822-5 (PMC12816672; doi:10.1038/s41467-025-66822-5)
Supplement: Supplementary file 1 — Supplementary Information [file 41467_2025_66822_MOESM1_ESM.pdf]

Supplementary Information

Global floating algae are expanding

Lin Qi[1,2], Menghua Wang[1], Brian B. Barnes[3], Douglas G. Capone[4], Joaquim I. Goes[5], Edward J. Carpenter[6], Yuyuan Xie[3], Chuanmin Hu[3*]

*Correspondence to: Chuanmin Hu, huc@usf.edu

This PDF file includes:

Supplementary Figures 1 to 9

Supplementary Figure 1. Flow chart showing the steps to map and quantify FA in global oceans.

Supplementary Figure 2. Architecture of the ResUNet DL model used in this study.

Supplementary Figure 3. Illustration of the "ground truth" image preparation.

Supplementary Figure 4. Training and test loss of the ResUNet DL model over epochs.

Supplementary Figure 5. Examples showing how the DL model and pixel classification worked for each main FA type identified in this study.

Supplementary Figure 6. Monthly climatology of global distributions of FA density between 2003 and 2022.

Supplementary Figure 7. Annual mean global distributions of FA density from 2003 to 2022

Supplementary Figure 8. Global distributions of several environmental variables that are important to *Trichodesmium* growth.

Supplementary Figure 9. Long-term changes of three types of macroalgae in their respective niche regions.

Supplementary Table 1

Supplementary Table 1. Evaluation of the ResUNet deep-learning (DL) model performance by FA type.

Source data are provided in the data repositories[1, 2] as well as in a Source Data file.

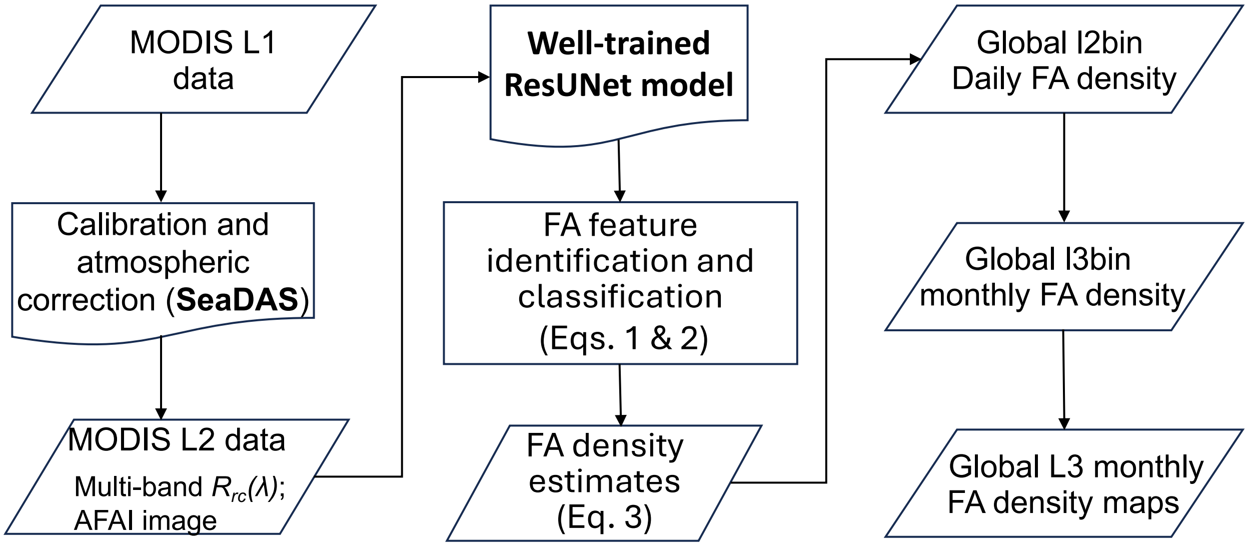

Supplementary Figure 1. Flow chart showing the steps to map and quantify FA in global oceans. Of these, the FA feature identification is through a well-trained DL model (ResUNet model), the FA classification is through examining the spectral shapes of the identified pixels (Eq. 7), and the FA areal density in each feature pixel is estimated using spectral unmixing (Eq. 8).

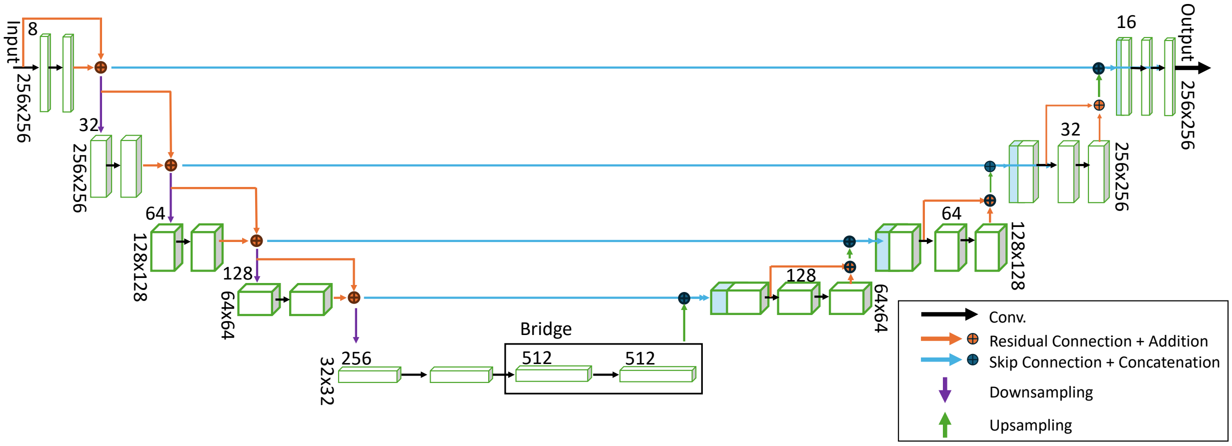

Supplementary Figure 2. Architecture of the ResUNet DL model used in this study. The network follows a U-shaped structure with an encoder, bridge, and decoder. Green cubes represent multichannel feature maps, with spatial dimensions (e.g., 256×256) indicated alongside and the number of channels labeled above each block. White cubes connected by dashed arrows represent skip connections used for feature concatenation. Orange arrows denote residual connections with element-wise addition, while blue arrows indicate skip connections with concatenation. Purple and green arrows represent downsampling and upsampling operations, respectively. Each convolutional layer is followed by batch normalization and a ReLU activation. The final segmentation output is produced through a sigmoid activation layer with 1 output channel (not shown).

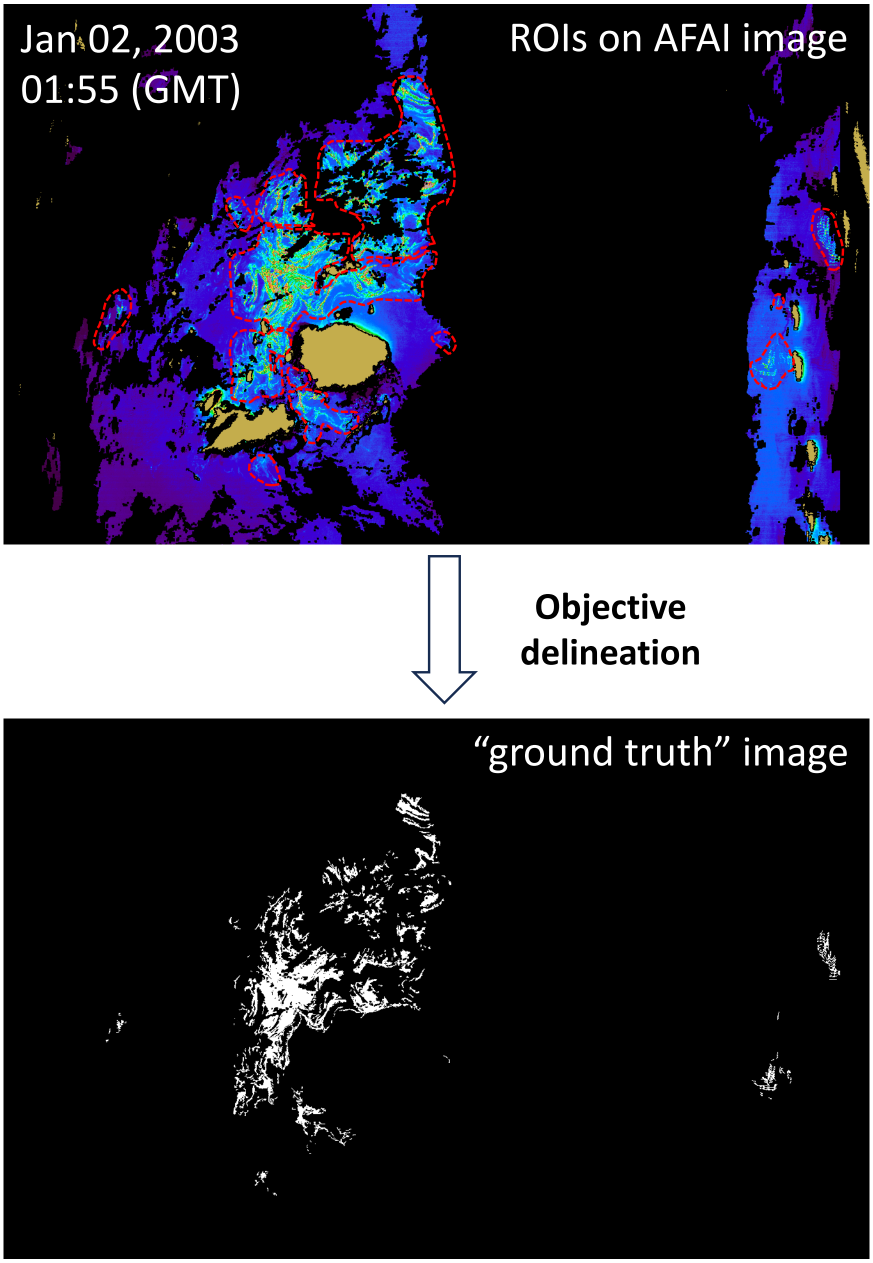

Supplementary Figure 3. Illustration of the “ground truth” image preparation. Regions of Interest (ROIs, red outlines) were manually selected on the AFAI image to exclude areas prone to false positives such as cloud edges, cloud shadows, nearshore shallow waters, and noise. Different thresholds were applied within each ROI to objectively delineate FA image features, resulting in the binary “ground truth” image. A total of 830 MODIS L2 “ground truth” images (6,912 sub-images) were prepared this way for DL model training and validation. Basemap generated using SeaDAS (https://seadas.gsfc.nasa.gov/). Source data are provided in the data repository[1].

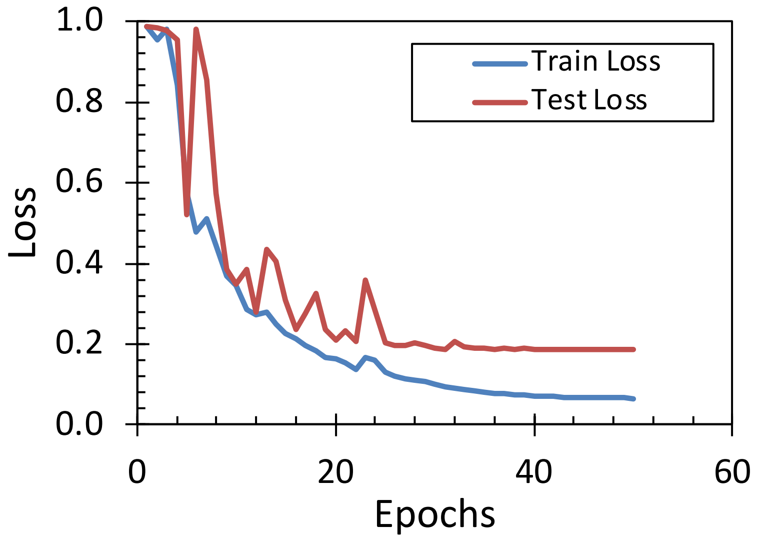

Supplementary Figure 4. Training and test loss of the ResUNet DL model over epochs. A total of 6,912 sub-images (256 × 256 × 8) were used as input with a batch size of 32. The loss decreased steadily during training and began to stabilize after approximately 30 epochs, indicating model convergence. Training was stopped at epoch 50 to avoid overfitting and maintain optimal performance.

Supplementary Figure 5 (part 1)

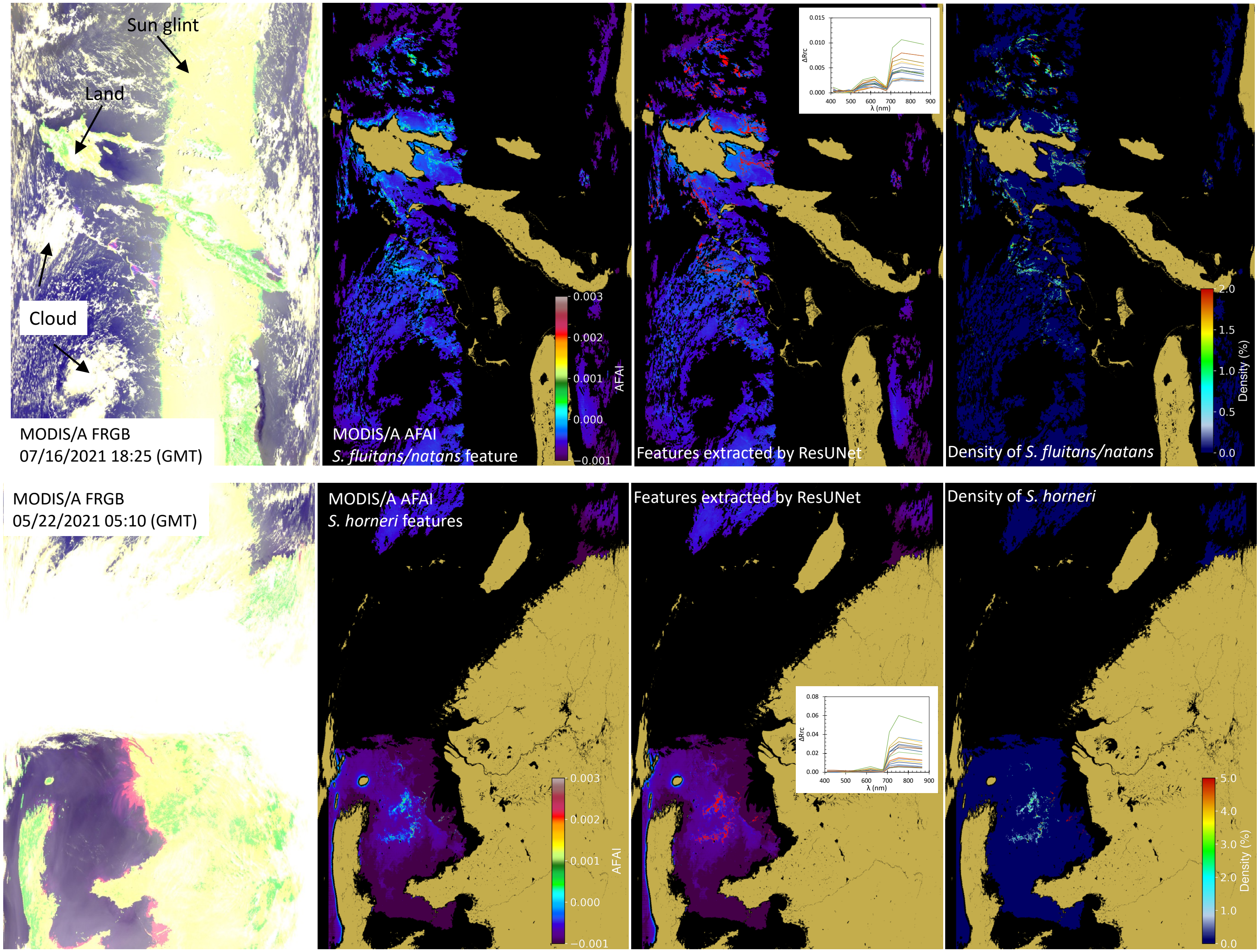

Supplementary Figure 5 (part 2)

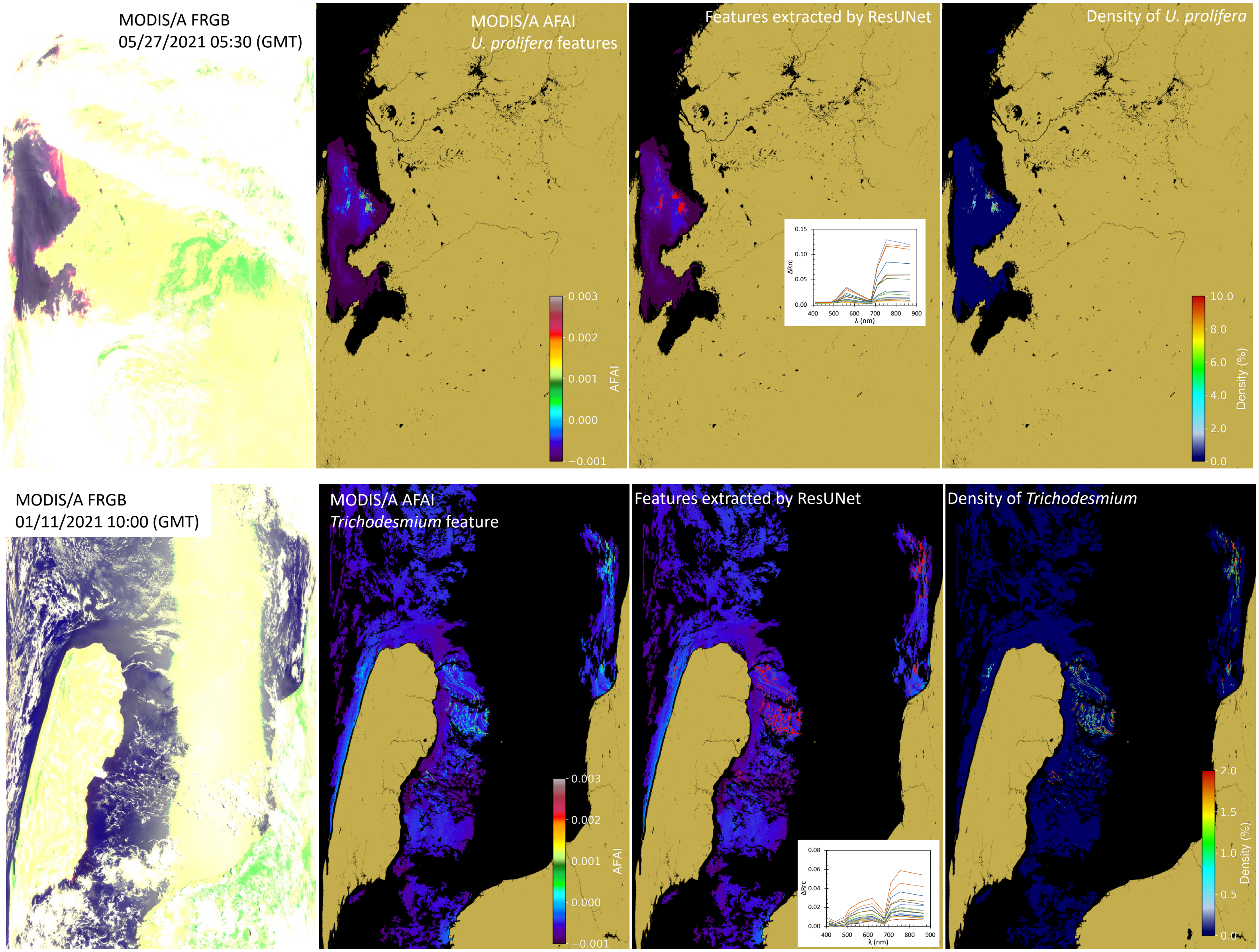

Supplementary Figure 5 (part 4)

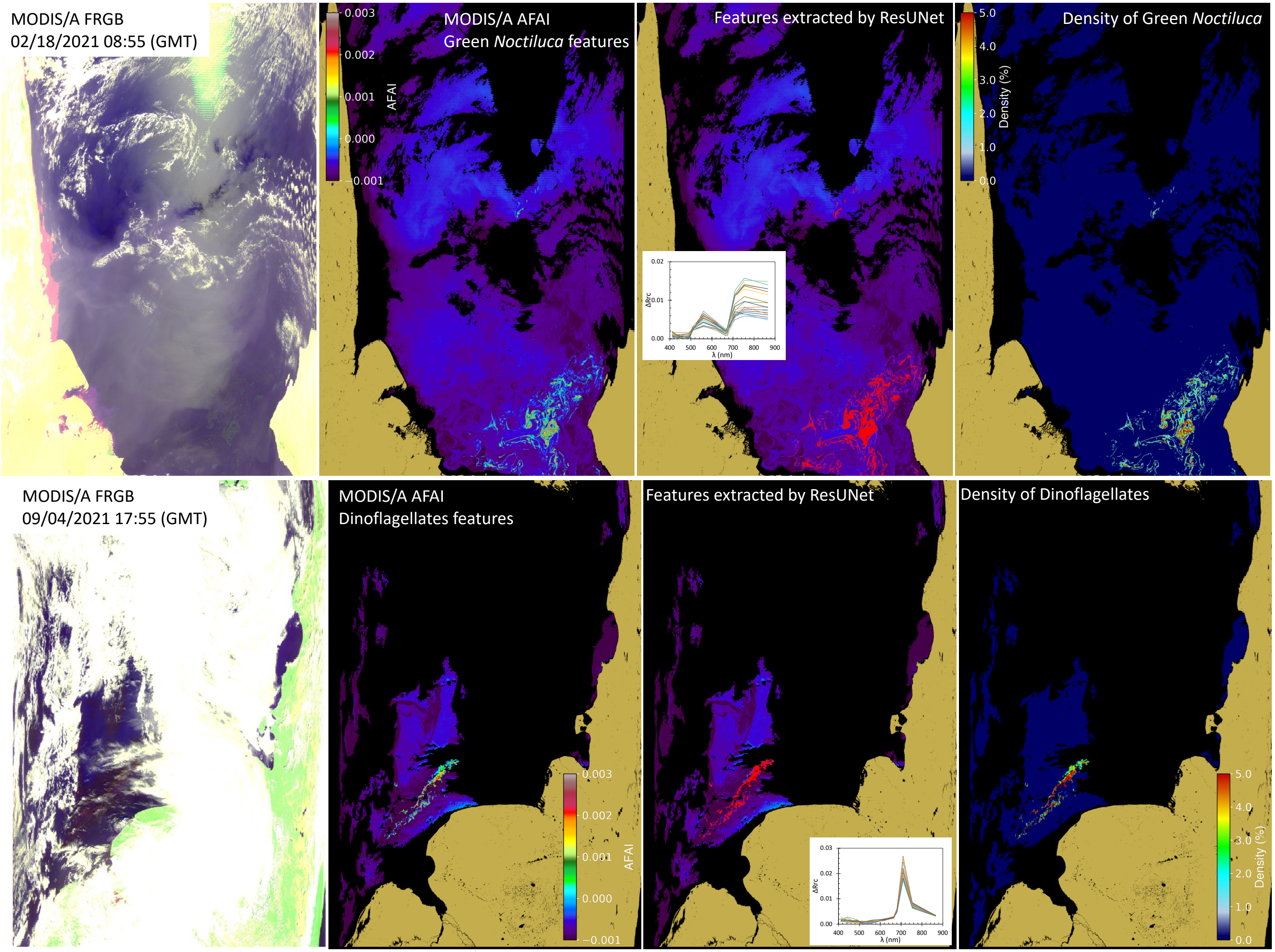

Supplementary Figure 5 (part 4)

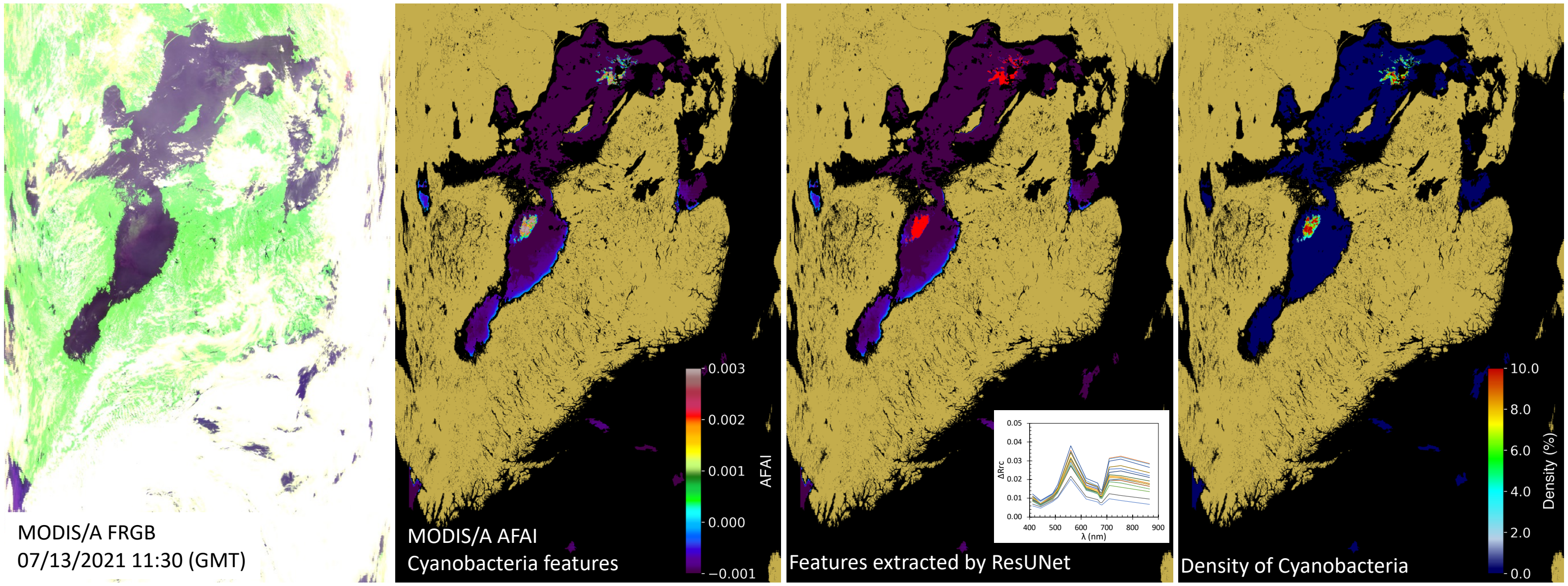

Supplementary Figure 5. Examples showing how the DL model and pixel classification worked for each main FA type identified in this study. First column: FRGB images showing land, water, and clouds; Second column: AFAI images showing elongated image features over water; Third column, DL model-extracted pixels (red) overlaid on the AFAI images; Last column: FA areal density (%) within each pixel; and the insert plots show a group of $\Delta R_{rc}(\lambda)$ spectra of randomly selected feature pixels for each type of FA. Basemap generated using SeaDAS (https://seadas.gsfc.nasa.gov/). Source data are provided in the data repository[1].

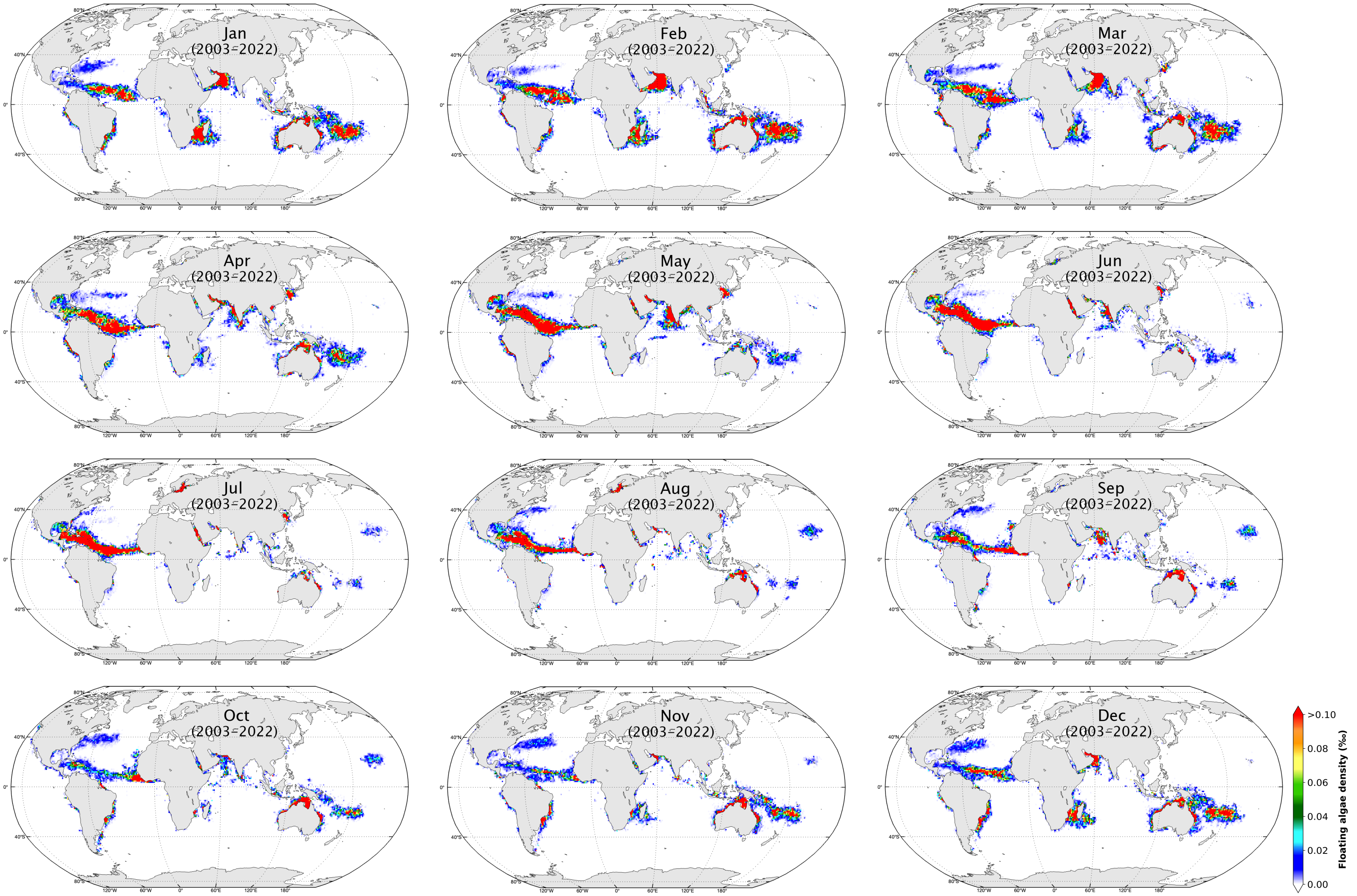

Supplementary Figure 6. Monthly climatology of global distributions of FA density between 2003 and 2022. Basemap generated using MATLAB M_Map package (www.eoas.ubc.ca/~rich/map.html). Source data are provided in the data repository[2].

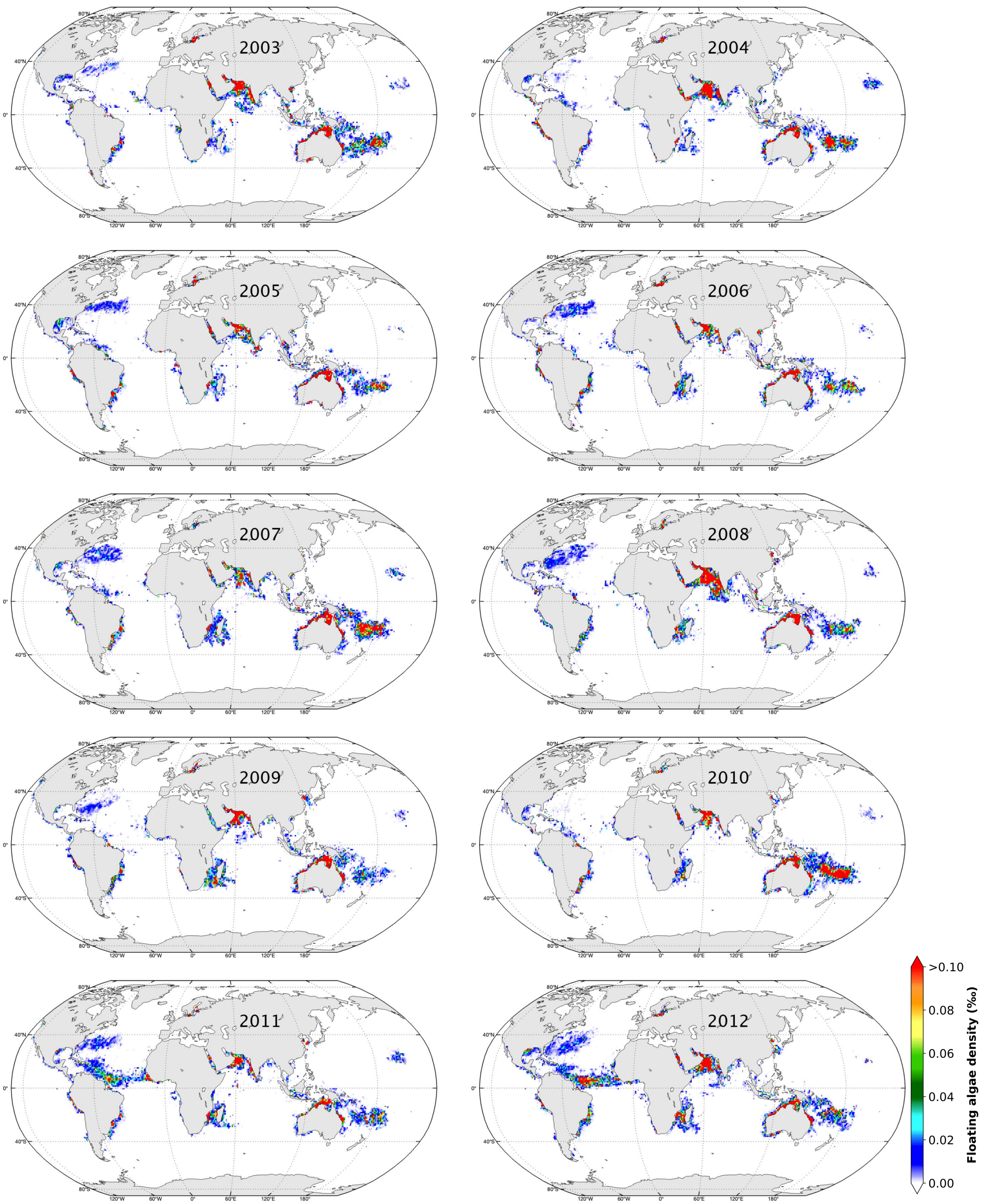

Supplementary Figure 7. Annual mean global distributions of FA density from 2003 to 2022 (continued on the next page). Basemap generated using MATLAB M_Map package (www.eoas.ubc.ca/~rich/map.html). Source data are provided in the data repository[2].

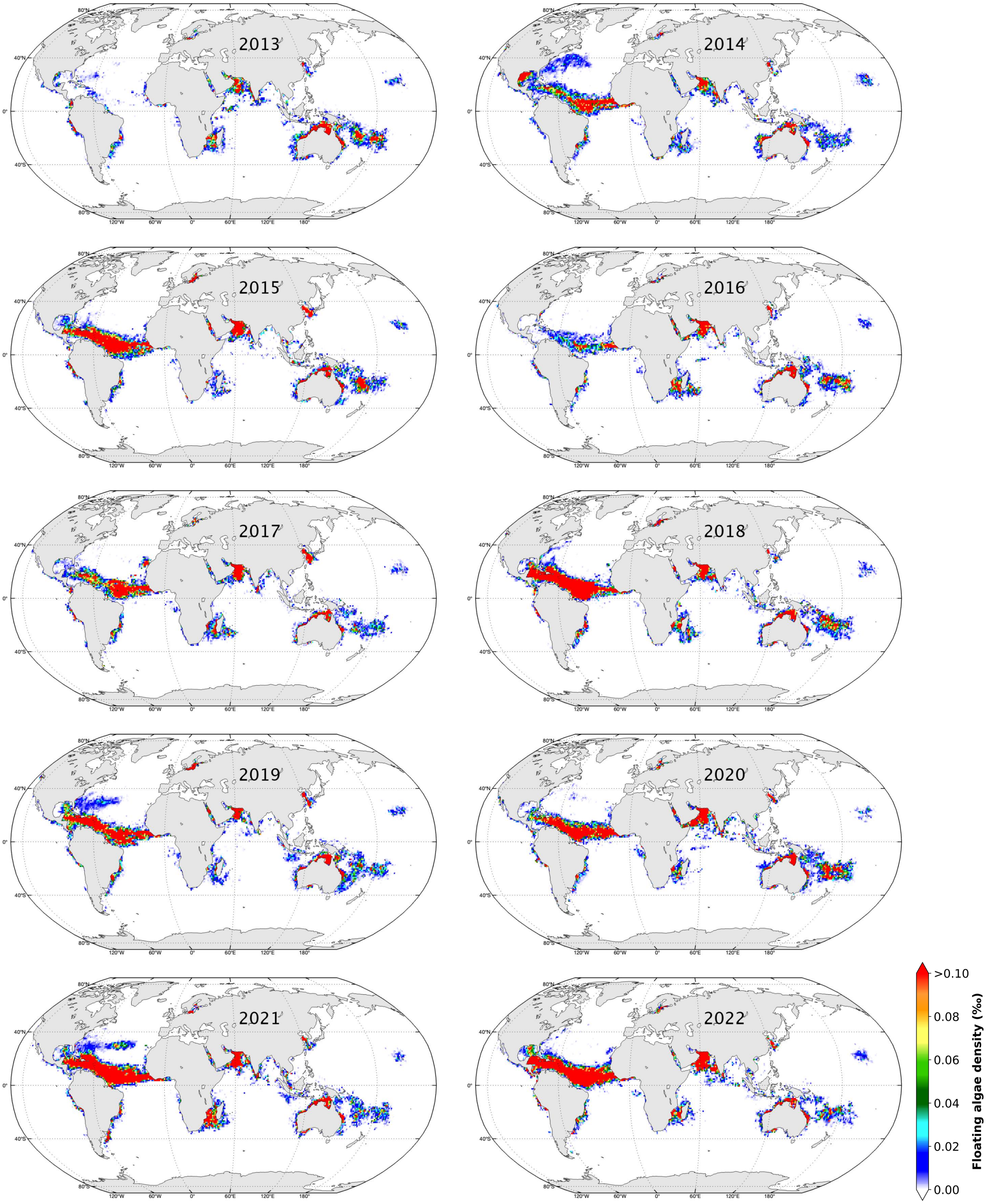

Supplementary Figure 7 (continued). Annual mean global distributions of FA density from 2003 to 2022. Basemap generated using MATLAB M_Map package (www.eoas.ubc.ca/~rich/map.html). Source data are provided in the data repository[2].

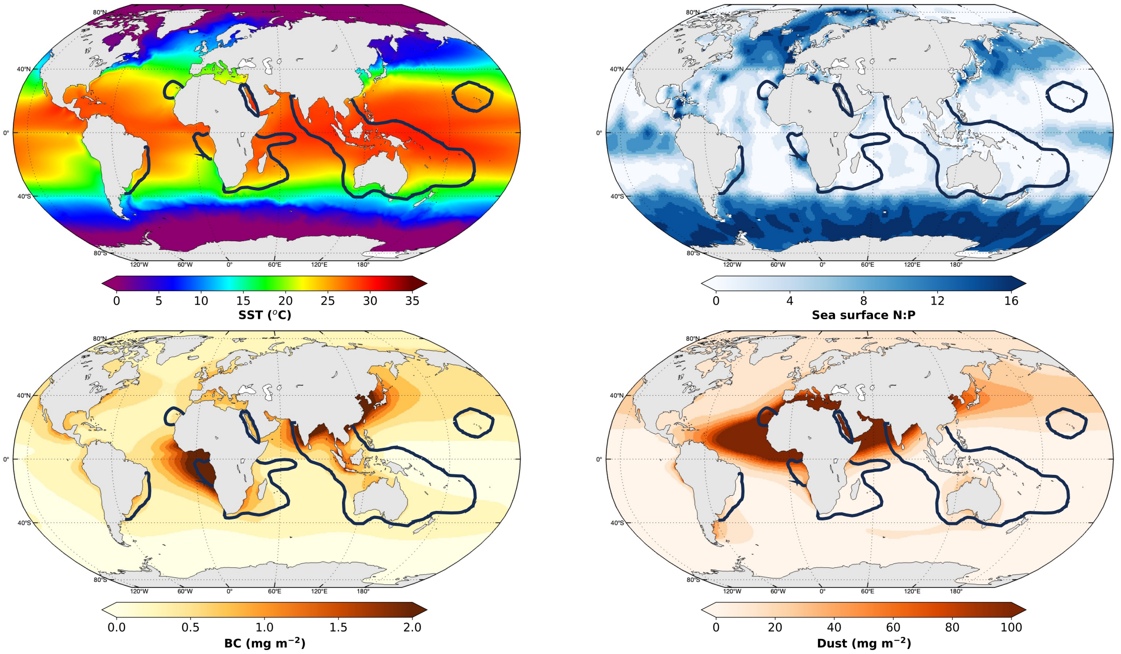

Supplementary Figure 8. Global distributions of several environmental variables that are important to *Trichodesmium* growth. Overlaid black lines on each panel are the *Trichodesmium* boundaries determined from this study (Fig. 1B). Basemap generated using MATLAB M_Map package (www.eoas.ubc.ca/~rich/map.html). Source data are provided by NASA and NOAA (https://disc.gsfc.nasa.gov/datasets/M2T1NXAER_5.12.4/summary; https://www.ncei.noaa.gov/access/world-ocean-atlas-2018/bin/woa18oxnu.pl)

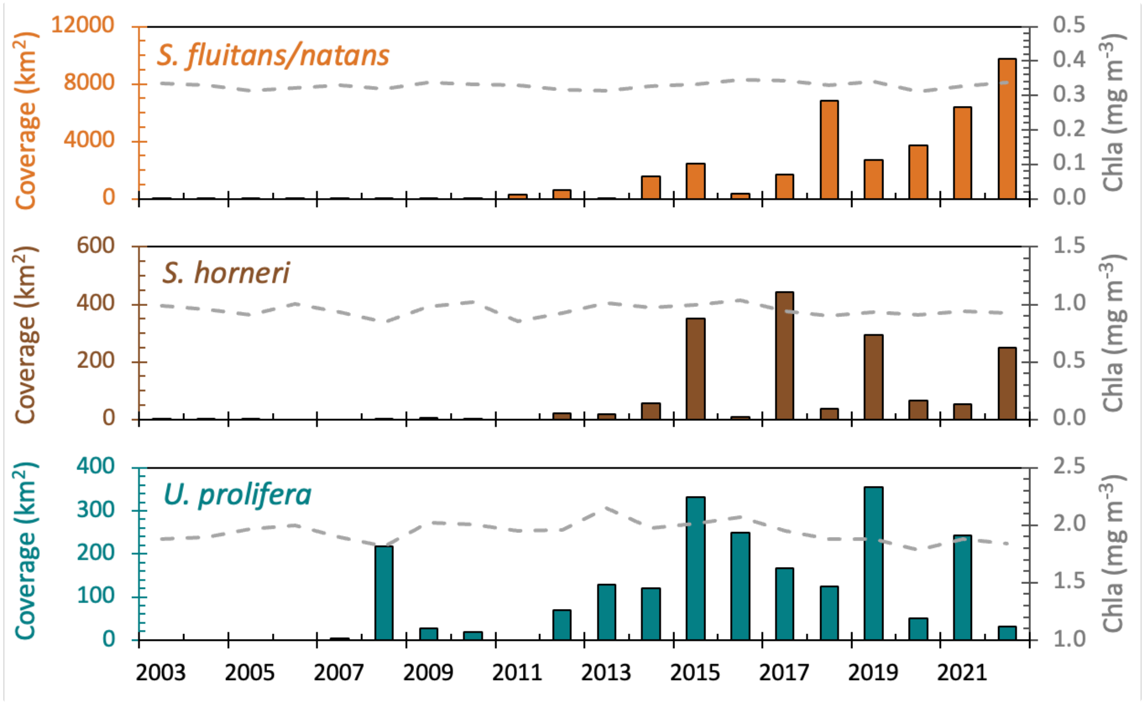

Supplementary Figure 9. Long-term changes of three types of macroalgae in their respective niche regions. For comparison, long-term changes in Chla concentrations are overlaid as dashed lines. Source data are provided in a Source Data file.

Supplementary Table 1. Evaluation of the ResUNet deep-learning (DL) model performance by FA type.

FA type	TP	FP	FN	Precision	Recall	F1 Score	IoU
	(weighted area in km^2)						
Microalgae	7011.3	406.9	633.7	94.5%	91.7%	93.1%	87.1%
Macroalgae	5065.0	445.4	536.5	91.9%	90.4%	91.2%	83.8%

Microalgae, also referred to as phytoplankton in this study, include *Trichodesmium*, green *Noctiluca*, cyanobacteria, and other dinoflagellates. Macroalgae (a.k.a seaweed), include *Sargassum fluitans/natans* in the Atlantic Ocean, *Sargassum horneri* in the East China Sea, and *Ulva prolifera* in the Yellow Sea. A total of 34 MODIS L2 images, 19 containing microalgae and 15 containing macroalgae, were used to evaluate the model performance. For definitions of the parameters, please see Methods.

Reference:

1 Qi, L. (2025). Deep Learning Model Training and Validation Data for Global Floating Algae Detection, University of South Florida, V1, https://doi.org/10.17632/f39zt9g2c4.1

2 Qi, L. & Hu, C. (2025). Global floating algae maps. figshare. Dataset. https://doi.org/10.6084/m9.figshare.28139492
