# Supplementary material for: Global floating algae blooms are expanding
Source: Nat Commun. 2025 Dec 7;17:612. doi: 10.1038/s41467-025-66822-5 (PMC12816672; doi:10.1038/s41467-025-66822-5)
Supplement: Supplementary file 3 — Reporting Summary [file 41467_2025_66822_MOESM3_ESM.pdf]

nature portfolio

Corresponding author(s): Chuanmin Hu

Last updated by author(s): 2025/08/04

Reporting Summary

Nature Portfolio wishes to improve the reproducibility of the work that we publish. This form provides structure for consistency and transparency in reporting. For further information on Nature Portfolio policies, see our Editorial Policies and the Editorial Policy Checklist.

Statistics

For all statistical analyses, confirm that the following items are present in the figure legend, table legend, main text, or Methods section.

n/a	Confirmed	
☐	☒	The exact sample size (*n*) for each experimental group/condition, given as a discrete number and unit of measurement
☒	☐	A statement on whether measurements were taken from distinct samples or whether the same sample was measured repeatedly
☐	☒	The statistical test(s) used AND whether they are one- or two-sided<br>*Only common tests should be described solely by name; describe more complex techniques in the Methods section.*
☒	☐	A description of all covariates tested
☒	☐	A description of any assumptions or corrections, such as tests of normality and adjustment for multiple comparisons
☐	☒	A full description of the statistical parameters including central tendency (e.g. means) or other basic estimates (e.g. regression coefficient) AND variation (e.g. standard deviation) or associated estimates of uncertainty (e.g. confidence intervals)
☐	☒	For null hypothesis testing, the test statistic (e.g. *F*, *t*, *r*) with confidence intervals, effect sizes, degrees of freedom and *P* value noted<br>*Give P values as exact values whenever suitable.*
☒	☐	For Bayesian analysis, information on the choice of priors and Markov chain Monte Carlo settings
☒	☐	For hierarchical and complex designs, identification of the appropriate level for tests and full reporting of outcomes
☒	☐	Estimates of effect sizes (e.g. Cohen's *d*, Pearson's *r*), indicating how they were calculated

Our web collection on statistics for biologists contains articles on many of the points above.

Software and code

Policy information about availability of computer code

Data collection	All data used in generating the global maps of floating algae were collected by satellites (MODIS on Aqua and OLCI on Sentinel-3A and Sentinel-3B). These are spectral radiance existing the atmosphere and captured by the satellite sensor.
Data analysis	The satellite data were processed using the NASA software SeaDAS (version 8.2, https://seadas.gsfc.nasa.gov), where the software package and its documentation can all be downloaded.<br>The ResUNet model used in this analysis to extract floating algae features from satellite imagery is available at GitHub (https://github.com/feevos/resuneta) but modified for this paper. Floating algae pixels are spectrally unmixed using computer codes developed in house.<br>The individual Level-2 data granules were "binned" to monthly global maps using NASA software SeaDAS (version 8.2), specifically its l3bin module. Time-series analysis was conducted using the Mann-Kendall test to determine Theil-Sen's slope and p-value using publicly available Python codes.

For manuscripts utilizing custom algorithms or software that are central to the research but not yet described in published literature, software must be made available to editors and reviewers. We strongly encourage code deposition in a community repository (e.g. GitHub). See the Nature Portfolio guidelines for submitting code & software for further information.

Data

Policy information about availability of data

All manuscripts must include a data availability statement. This statement should provide the following information, where applicable:

- Accession codes, unique identifiers, or web links for publicly available datasets
- A description of any restrictions on data availability
- For clinical datasets or third party data, please ensure that the statement adheres to our policy

All satellite data used in this analysis is available through the NASA OB.DAAC (https://oceancolor.gsfc.nasa.gov). The calibrated radiance data have been made available by the U.S. NASA through OB.DAAC (https://oceancolor.gsfc.nasa.gov), where full documentation on how data are preprocessed, data format, file format, and other ancillary information are available. In total, about 1.2 million MODIS granules were downloaded and analyzed. The environmental data used in this analysis is available through their specific data providers as described in the Materials and Methods section. All satellite data used in the training and validation of the deep learning model to detect floating algae from MODIS images has been made available through a public data repository (https://doi.org/10.17632/f39zt9g2c4.1), including the spectral reflectance data, quick-look images, "ground truth" images, and model output images. All monthly maps of floating algae have been made available through the figshare data repository (https://doi.org/10.6084/m9.figshare.28139492). All source data used to generate the time-series plots are made available through a Source Data file.

Research involving human participants, their data, or biological material

Policy information about studies with human participants or human data. See also policy information about sex, gender (identity/presentation), and sexual orientation and race, ethnicity and racism.

Reporting on sex and gender	N/A
Reporting on race, ethnicity, or other socially relevant groupings	N/A
Population characteristics	N/A
Recruitment	N/A
Ethics oversight	N/A

Note that full information on the approval of the study protocol must also be provided in the manuscript.

Field-specific reporting

Please select the one below that is the best fit for your research. If you are not sure, read the appropriate sections before making your selection.

☐ Life sciences ☐ Behavioural & social sciences ☒ Ecological, evolutionary & environmental sciences

For a reference copy of the document with all sections, see nature.com/documents/nr-reporting-summary-flat.pdf

Ecological, evolutionary & environmental sciences study design

All studies must disclose on these points even when the disclosure is negative.

Study description	The study is based nearly entirely on satellite data collected by MODIS and OLCI sensors, which measure the reflected sun light. Such data carry information on floating plants (macrolagae mats and microalgae scums) on the ocean surface, which is analyzed to generate global maps of floating algae distributions, from which their spatial patterns and temporal changes are analyzed. The most striking result is the disparity between macroalgae and microalgae, with the former showing rapid increases in the recent decade where a regime shift in the global ocean may have occurred.
Research sample	All satellite images collected by MODIS between 2003 and 2022 were used in this analysis. The total number of data files is about 1.2 million. Each data file contain reflected sunlight (total radiance) in multiple spectral bands, captured by the satellite sensors, which was vicariously calibrated by the NASA and ESA science teams.
Sampling strategy	To assure statistically meaning results, ALL satellite data collected during the study period were used without subsampling.
Data collection	Data were collected by MODIS and OLCI on polar-orbiting satellites. These satellites orbit the earth following the sun, which capture reflected sunlight around 1.30pm and 11:00 am local time respectively. The data were collected continuously along the orbits, with a revisit frequency being 1-2 days (for MODIS) and 2-3 days (for OLCI). Data have been processed and stored at NASA OB.DAAC (https://oceancolor.gsfc.nasa.gov), where full documentation on data collection and processing as well as on file structure is available.
Timing and spatial scale	The data covered the time period of 2003-2022 and the entire globe, with a spatial resolution of about 1 km per image pixel.
Data exclusions	No data was excluded in this analysis.

Reproducibility	All data were generated by NASA and available at OB.DAAC (https://oceancolor.gsfc.nasa.gov) to all researchers. The computer codes used to extract features are available through GitHub and through previous publications. All model training and validation data files as well as their README files have been uploaded to a public data repository (https://doi.org/10.17632/f39zt9g2c4.1) for others to test and reproduce.
Randomization	For the deep-learning model development, satellite images were randomly split into two groups, one for model training and the other for model validation. Further details are provided in the Methods section and Supplemental Information of this manuscript.
Blinding	The split of the satellite images in to training and validation groups was purely random without human intervention.

Did the study involve field work? ☐ Yes ☒ No

Reporting for specific materials, systems and methods

We require information from authors about some types of materials, experimental systems and methods used in many studies. Here, indicate whether each material, system or method listed is relevant to your study. If you are not sure if a list item applies to your research, read the appropriate section before selecting a response.

Materials & experimental systems

n/a	Involved in the study
☒	☐ Antibodies
☒	☐ Eukaryotic cell lines
☒	☐ Palaeontology and archaeology
☒	☐ Animals and other organisms
☒	☐ Clinical data
☒	☐ Dual use research of concern
☒	☐ Plants

Methods

n/a	Involved in the study
☒	☐ ChIP-seq
☒	☐ Flow cytometry
☒	☐ MRI-based neuroimaging

Plants

Seed stocks	N/A
Novel plant genotypes	N/A
Authentication	N/A
